# NLRP3 Inflammasomes: Central Players and Therapeutic Targets in Alzheimer's disease Pathogenesis

**DOI:** 10.1002/alz70855_104829

**Published:** 2025-12-23

**Authors:** Chirag Sharma

**Affiliations:** ^1^ MMCP, Ambala, Haryana, India

## Abstract

**Background:**

Neurodegenerative illness has become much more common in recent years, and this trend is predicted to continue as the world's population ages quickly. Protein aggregation, metabolic disorders, immunological dysregulation, and a gradual loss of neurons in the brain or peripheral nervous system are the hallmarks of these diseases.

**Method:**

The nucleotide‐binding domain leucine‐rich repeat and pyrin domain containing receptor protein 3 (NLRP3) is a crucial pattern recognition receptor in human innate immunity. It is largely responsible for triggering elements such as reactive oxygen species, lysosomal damage, and mitochondrial DNA, ultimately leads to Alzheimer's disease (AD) ‐ like pathology. Furthermore, several reported studies from literature supported this phenomenon. Additionally, by generating cytokines and chemokines such as IL‐1β, IL‐18, and other subsequently leads to the deposition of tau and Aβ proteins (pathological hallmark of AD) and caused AD development. Further, certain mechanisms, including autophagy, receptor binding blocking, and aberrant cytokine release, have been found to have a detrimental impact on NLRP3 inflammasome activation.

**Result:**

From these insights we were able to summarize that the most recent advancements in AD prevention and treatment involve NLRP3 inflammasome as well as above mentioned possible mechanisms involved in AD pathogenesis.

**Conclusion:**

By targeting the activation or inactivation of the NLRP3 inflammasome, it could be possible to reveal the pathophysiology of AD from a new perspective and provide a novel strategy for its prevention and treatment.

**Keywords**: AD**(Alzheimer's disease),** NLRP3 inflammasome, interleukins, Cytokines, Chemokines, Tau protein, leucine, neurodegenerative diseases.

